# Genetic polymorphism of *SLC31A1* is associated with clinical outcomes of platinum-based chemotherapy in non-small-cell lung cancer patients through modulating microRNA-mediated regulation

**DOI:** 10.18632/oncotarget.24794

**Published:** 2018-05-08

**Authors:** Chang Sun, Zhuojun Zhang, Jingbo Qie, Yi Wang, Ji Qian, Jiucun Wang, Junjie Wu, Qiang Li, Chunxue Bai, Baohui Han, Zhiqiang Gao, Jibin Xu, Daru Lu, Li Jin, Haijian Wang

**Affiliations:** ^1^ State Key Laboratory of Genetic Engineering and Collaborative Innovation Center for Genetics and Development, School of Life Sciences, Fudan University, Shanghai, China; ^2^ Department of Respiratory and Critical Care Medicine, Changhai Hospital, the Second Military Medical University, Shanghai, China; ^3^ Department of Pulmonary Medicine, Zhongshan Hospital of Fudan University, Shanghai, China; ^4^ Department of Pneumology, Chest Hospital, Shanghai Jiaotong University, Shanghai, China; ^5^ Department of Cardiothoracic Surgery, Changzheng Hospital of the Second Military Medical University, Shanghai, China

**Keywords:** NSCLC, pharmacogenetics, SLC31A1, SNP, microRNA

## Abstract

SLC31A1 is the major transporter for platinum drug intake, its expression correlates with drug disposition and response. In 1004 Chinese NSCLC patients with platinum-based chemotherapy, we investigated the association between *SLC31A1* polymorphisms and clinical outcomes. Heterozygotes of rs10759637 at 3′UTR was associated with severe thrombocytopenia (odds ratio [OR]: 2.69; *P* = 0.012) and shorter overall survival (hazard ratio [HR]: 1.24; *P* = 0.005). Variant homozygote of rs2233914 was correlated with longer overall survival (hazard ratio [HR]: 0.73; *P* = 0.008). Haplotype and diplotype of these linked SNPs were associated with hematologic toxicities. In stratification analyses, rs10759637 and rs2233914 consistently correlated with overall survival in specific subgroups such as men, smoker, patients older than 58 years, or with ECOG PS 0-1, or with squamous cell carcinoma. rs10759637 could change the local structure of 3′UTR harboring putative binding sites for hsa-miR-29, whose transfection into 16HBE cells resulted in remarkable suppression of gene expression. The rs10759637 variant significantly correlated with lowered luciferase activity in reporter assays and decreased expression of *SLC31A1* transcript in tumorous tissues. The study thereby identified functional polymorphism of *SLC31A1* that modulates miRNA-3′UTR interaction and gene expression as potential pharmacogenetic biomarker for clinical outcomes of platinum-based chemotherapy in NSCLC patients.

## INTRODUCTION

Lung cancer is among the most common cancers in both man and woman, and is the leading cause of cancer-related death worldwide [1]. Non-small cell lung cancer (NSCLC), with two main histological types of adenocarcinoma and squamous-cell (epidermoid) carcinoma, accounts for about 80% of primary lung cancer, and a majority present with advanced stage (III/IV) when they were first diagnosed. Only 10–15% newly diagnosed NSCLC patients were adopted with a potentially curative resection [2]. Despite the introduction of targeted therapy on specific mutant molecules such as EGFR, platinum agents combined with another cytotoxic compound are still the first-line chemotherapy for advanced NSCLC. The action mode of platinating agents is known to bind the DNA with intra- or interstrand crosslinks and the Pt-DNA adduct lead to DNA lesions, activation of multiple pathways and result in cell apoptosis ultimately [3]. Despite efforts to improve therapeutic efficacy, the platinum-based chemotherapy brings modest benefits but also adverse effects, with the five-year survival rates less than 15%. And an increasing evidence showed that the limited efficacy for advanced NSCLC is due to drug resistance and toxicological side effects such as thrombocytopenia, nausea/vomiting, ototoxicity, nephrotoxicity and peripheral neurotoxicity, etc [3].

Mechanistically, therapeutic efficacy and response to platinum-based chemotherapy could be linked to these biological pathways, altered cellular accumulation, cytosolic inactivation of platinum drugs, inactivation of DNA repair pathway, altered apoptosis or increased tolerance to DNA damage [3]. The aberrant regulation and dysfunction of candidate genes in these pathway, which are largely ascribed to their functional polymorphisms, may influence interindividual differences in clinical outcomes. Thus there is a growing need toward tailoring chemotherapy to identify these functional genetic polymorphisms as predictive pharmacogenetic markers for better efficacy and minimal toxicity. To this end, the influence of sequence variants in pharmacodynamics pathways, such as nucleotide excision repair pathway, on clinical outcome has been extensively investigated [4]. In contrast, the biological function and clinical relevance of genetic polymorphisms in pharmacokinetics pathway for platinum metabolism and disposition are largely elusive.

Human SLC31A1 (solute carrier family 31 member 1) gene, also known as CTR1 (copper transporter 1), encodes a high-affinity copper transporter in cell membrane that functions as a homotrimer to effect the uptake of dietary copper. Recently, an interesting possibility has clearly emerged that the copper transporter SLC31A1 also acts as the major plasma-membrane transporter for platinum drug intake, including cisplatin, oxaliplatin and carboplatin [5]. Deletion of Slc31a1 in yeast and murine cells results in reduced cisplatin accumulation and increased resistance [6]. On the contrary, transfection of small cell lung cancer cell lines with SLC31A1 gene correlates with enhanced uptake of carboplatin and oxaliplatin [7]. Consistently, Slc31a1 knockout in murine model has also been shown to completely eliminate cisplatin tumor response *in vivo* [8]. Furthermore, in clinical setting, NSCLC patients with undetectable SLC31A1 expression in their tumors had reduced platinum concentration and tumor response, and lower platinum concentration in clinical specimens correlates directly with reduced tumor response and shorter survival time [9, 10]. Recently, in two pilot studies with relatively small sample size (two to three hundreds) in Chinese patients with NSCLC who received Pt-based therapy, Xu *et al.* documented that *SLC31A1* polymorphisms are associated with Pt-resistance or toxicity and poor clinical outcomes [11, 12]. But these results have not been validated in larger cohort of patients and functional implication of these associated variants are not clear.

In the present study, we assessed the association of tagging and potentially functional SNPs of *SLC31A1* gene with toxicological phenotypes, objective response and survivals of 1004 Chinese NSCLC patients receiving platinum-based treatment. We also functionally characterized a common variant at 3′ untranslated region (3′UTR) that modulates the microRNA-3′UTR interaction and decreases gene expression, proposing a possible underlying mechanism for the genetic association of *SLC31A1* with clinical outcomes.

## RESULTS

### Patient characteristics and clinical outcomes

A total of 1004 eligible NSCLC patients of Chinese population were recruited in the study to investigate the genetic association between *SCL31A1* polymorphisms and clinical outcomes of platinum-based chemotherapy. The main characteristics and clinical outcomes of patients are summarized in Table 1. Ever smokers account for 57.5% of the patients. Adenocarcinoma was the most common histological type (62.9%). Severe gastrointestinal toxicity (nausea/vomiting) was observed in 8.3% of the evaluated patients (*n* = 964). Severe hematological toxicity was observed in 23.9% of the evaluated patients (*n* = 969), among which 29 (3.1%), 149 (15.2%), 115 (12.3%) and 34 (3.6%) patients suffered from grade 3 or 4 anemia, leukopenia, neutropenia, thrombocytopenia, respectively. Grade 3 or 4 overall toxicity was observed in 29.9% of the evaluated patients (*n* = 952). For survival analysis, by the time of final data collection (July 2012), the median follow-up time was 46.5 month, and death had occurred in 74.9% of enrolled patients. The median progression-free survival (PFS) was 9.1 months, and the median overall survival (OS) was 19.3 months. The rates of clinical phenotypes such as toxicities in our study were quite comparable to those previously reported in large randomized clinical trials [13].

**Table 1:** Patient characteristics and clinical outcomes

Characteristic	Total Number	Number	Percent
All patients	1004		
Sex	1004		
Male		706	70.3
Female		298	29.7
Age	1004		
≤58		518	51.6
>58		486	48.4
Smoking Status	1000		
Ever Smoker		575	57.5
Nonsmoker ^*a*^		425	42.5
ECOG performance status ^*b*^	990		
0–1		904	91.3
2		86	8.7
TNM stage	999		
IIIA		81	8.1
IIIB		293	29.3
IV		625	62.6
Histological type	1004		
Adenocarcinoma (AC)		632	62.9
Squamous cell carcinoma (SCC)		221	22.1
Adenosquamocarcinoma		20	2.0
Others ^*c*^		131	13.0
Chemotherapy regimens	1004		
Platinum (cisplatin)-navelbine		316	31.5
Platinum (cisplatin)-gemcitabine		239	23.8
Platinum (carboplatin)-paclitaxel		313	31.2
Platinum-docetaxel		87	8.7
Other platinum combinations		49	4.9
Objective response	976		
Complete response (CR)		1	0.1
Partial response (PR)		176	18.0
Stable disease (SD)		611	62.6
Progressive disease (PD)		188	19.3
Toxicity outcome			
Grade 3 or 4 gastrointestinal toxicity			
Nausea/vomiting	964	80	8.3
Grade 3 or 4 hematologic toxicity	969	232	23.9
Anemia	944	29	3.1
Leukopenia	980	149	15.2
Neutropenia	935	115	12.3
Thrombocytopenia	950	34	3.6
Grade 3 or 4 overall toxicity	952	285	29.9
Median time to outcomes (months)	972		
Progression-free survival (PFS)		9.1	
Overall survival (OS)		19.3	

^*a*^ Nonsmokers were defined as those who had smoked <1 cigarette per day and for <1 year in their lifetime.

^*b*^ ECOG PS, Eastern Cooperative Oncology Group performance status.

^*c*^ Other carcinomas included mixed cell or undifferentiated carcinoma.

**Figure 1:**
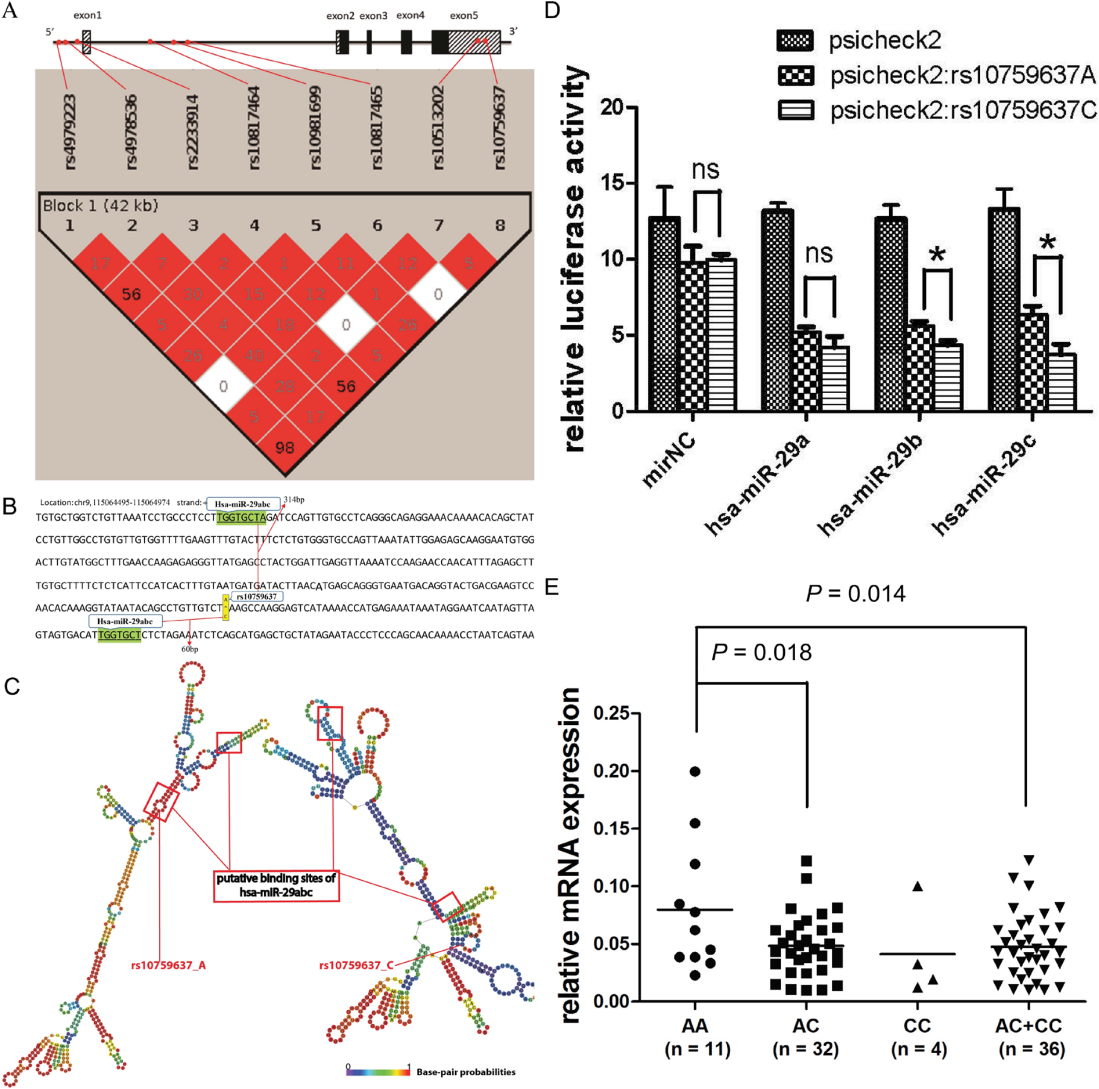
rs10759637 in *SLC31A1* 3′UTR decreases microRNA-medicated gene expression (**A**) Schematic structure of *SLC31A1* gene and linkage disequilibrium of its candidate SNPs (color intensity is proportional to *D*′ and percent numbers represent *r*^2^). (**B**) Local sequence of *SLC31A1* 3′UTR annotated with the close locations of rs10759637 and two putative binding sites for hsa-miR-29 family. (**C**) Comparison of secondary structures, built with RNAfold program, of the local *SLC31A1* 3′UTRs harboring hsa-miR-29 binding sites and the wild or the variant alleles of rs10759637. (**D**) Luciferase reporter assays in 16HBE cells to analyze the regulatory role hsa-miR-29 on *SLC31A1* 3′UTR that could be modulated by rs10759637. Co-transfection with hsa-miR-29 member (a, b, or c) and *SLC31A1* 3′UTR construct consistently resulted in reduced luciferase activity. In the cases of hsa-miR-29 b or c, rs10759637 allelic state significantly affected the *cis*-regulation toward reporter expression. (**E**) Real-time quantitative RT-PCR analysis of *SLC31A1* mRNA expression in lung tumorous tissues from an independent cohort of patients, which was significantly correlated with rs10759637 genotypes.

In the NSCLC patient cohort, we genotyped eight tagging and potentially functional SNPs of *SLC31A1* gene, three at 5′flanking region (rs4979223, rs4978536 and rs2233914), three at the intron (rs10817464, rs10981699 and rs10817465), and two at 3′UTR (rs10513202 and rs10759637), all of which were in Hardy-Weinberg equilibrium (HWE) (Supplementary Table 1), and six of which are common variants (minor allele frequency, MAF >0.05) in this study population. The linkage disequilibrium analysis showed that the eight candidate SNPs could been partitioned into one haplotype block. For example, rs10759637 at 3′UTR are strictly associated with rs4979223 (*r*^2^ = 0.98) and rs2233914 (*r*^2^ = 0.56) at 5′flanking region (Figure 1A).

### Association of SLC31A1 gene polymorphisms with toxicity and objective response

In the patient cohort that had been evaluated for the seven toxicological phenotypes after platinum-based chemotherapy, we compared the genotypic distributions for the eight candidate SNPs of *SLC31A1* between groups with respective mild or severe toxicological outcomes (Supplementary Table 2). Interestingly, we observed that rs4979223 (*P* = 0.007), rs4978536 (*P* = 0.063, with marginal significance), rs2233914 (*P* = 0.039), rs10817464 (*P* = 0.006) and rs10759637 (*P* = 0.006) showed significantly divergent genotypic distribution between groups with mild or severe thrombocytopenia, some of which remained statistically significant after multiple test correction. Based on these five candidate SNPs, we then assessed the association between *SLC31A1* polymorphism and toxicological outcomes with multivariate unconditional logistic regression analysis (Table 2). As to the tightly linked rs4979223 and rs10759637, we found their heterozygous genotype (A/C), as compared to the grouped homozygous genotypes (A/A+C/C), were significantly associated with severe thrombocytopenia, with ORs (95% CI) being 2.60 (1.20−5.64) and 2.69 (1.24−5.83), respectively. The variant containing genotype group (A/G+G/G) of rs4978536, as compared to the wild A/A, was also associated with severe thrombocytopenia (OR 2.59; 95% CI 1.23−5.47). In alike manner, the variant genotypes of rs10817464 were associated with increased risks of severe thrombocytopenia (OR 3.09; 95% CI 1.20−7.93), leucopenia (OR 2.16; 95% CI 1.19−3.92), hematologic toxicity (OR 1.98; 95% CI 1.19−3.29) and overall toxicity (OR 1.98; 95% CI 1.21−3.22). On the contrary. The variant A/A genotype of rs2233914, as referred to the wild allele containing genotypes (G/G+G/A), was associated with mild overall toxicity (OR 0.59; 95% CI 0.36−0.95).

**Table 2:** Association between *SLC31A1* SNPs and toxicity outcomes

Reference SNP	Genotype	Toxicity	Toxicity grade (G3-4/G0-2)	*P* value^a^	OR (95% CI)^b^	*P* value^b^
rs4979223	A/A	Thrombocytopenia	3/282	0.007 ^*c*^	1.00 (ref)	
	A/C		24/417		**4.90 (1.43−16.82)**	**0.012**
	C/C		7/214		3.00 (0.75−12.05)	0.121
	A/C *vs* A/A+C/C		10/496	0.004 ^*c*^	**2.60 (1.20−5.64)**	**0.015**
rs4978536	A/A	Thrombocytopenia	20/701	0.063	1.00 (ref)	
	A/G		13/195		**2.71 (1.26−5.81)**	**0.010**
	G/G		1/20		1.63 (0.19−13.89)	0.654
	A/G+G/G *vs* A/A		14/215	0.018	**2.59 (1.23−5.47)**	**0.012**
rs2233914	G/G	Overall toxicity	135/299	0.150	1.00 (ref)	
	G/A		124/277		0.88 (0.64−1.20)	0.409
	A/A		26/91		**0.55 (0.33−0.91)**	**0.020**
	A/A *vs* G/G+G/A		259/576	0.052	**0.59 (0.36−0.95)**	**0.029**
rs10817464	A/A	Leucopenia	130/767	0.069	1.00 (ref)	
	A/G		19/62		**2.21 (1.22−4.01)**	**0.009**
	G/G		0/2		NA	NA
	A/G+G/G *vs* A/A		19/64	0.041	**2.16 (1.19−3.92)**	**0.011**
	A/A	Thrombocytopenia	27/842	0.006 ^*c*^	1.00 (ref)	
	A/G		6/73		2.75 (1.02−7.42)	0.045
	G/G		1/1		13.70 (0.69−220.29)	0.085
	A/G+G/G *vs* A/A		7/74	0.024	**3.09 (1.20−7.93)**	**0.019**
	A/A	Hematologic toxicity	204/683	0.088	1.00 (ref)	
	A/G		27/53		**1.92 (1.15−3.21)**	**0.012**
	G/G		1/1		6.34 (0.36−110.34)	0.205
	A/G+G/G *vs* A/A		28/54	0.024	**1.98 (1.19−3.29)**	**0.008**
	A/A	Overall toxicity	251/619	0.050	1.00 (ref)	
	A/G		33/47		**1.95 (1.19−3.19)**	**0.008**
	G/G		1/1		3.62 (0.20−66.75)	0.387
	A/G+G/G *vs* A/A		34/48	0.017	**1.98 (1.21−3.22)**	**0.006**
rs10759637	A/A	Thrombocytopenia	3/286	0.006 ^*c*^	1.00 (ref)	
	A/C		24/414		**5.10 (1.49−17.52)**	**0.010**
	C/C		7/216		3.05 (0.76−12.24)	0.116
	A/C *vs* A/A+C/C		10/502	0.004 ^*c*^	**2.69 (1.24−5.83)**	**0.012**

^*a*^ *P* values of Pearson χ^2^ tests for overall distributions for SNPs genotypes between severe and mild toxicological phenotypes.

^*b*^ Odds ratios (OR) and their 95% confidence intervals (CIs) and *P* values were calculated with unconditional logistic regression analysis, with adjustment of gender, age, smoking status, ECOG performance status, TNM status, histological types, and treatment regimen.

^*c*^ Statistical significance remained after multiple tests adjustment taking into account linkage disequilibrium between polymorphisms.

Because these five candidate SNPs of *SLC31A1* could be partitioned into one haplotype block (Figure 1), we also estimated their haplotype and diplotype frequencies in the cohort, and analyzed their association with toxicological outcomes (Table 3). We predicted only four common haplotypes for the five SNPs in the 1004 individuals, which is consistent with their strong linkage disequilibrium. Agreeing with the genotype-based association results as above, the Hap4_CGGGC, which is composed of rs4979223-variant-C, rs4978536-variant-G, rs2233914-wild-G, rs10817464-variant-G and rs10759637-variant-C alleles, was significantly associated with severe toxicological outcomes such as leucopenia (OR 1.88; 95% CI 1.04−3.40), thrombocytopenia (OR 2.81; 95% CI 1.19−6.60), hematologic toxicity (OR 1.88; 95% CI 1.14−3.08) and overall toxicity (OR 1.89; 95% CI 1.17−3.06). As compared to non-Hap4 carrier diplotype, the Hap4 carrier diplotype was also consistently associated with these severe toxicological phenotypes, with ORs (95% CI) being 2.16 (1.19−3.92), 3.09 (1.20−7.93), 1.98 (1.19−3.29) and 1.98 (1.21−3.22), respectively.

**Table 3:** Association between *SLC31A1* haplotype and diplotype and toxicity outcomes

Haplotype or Diplotype a	Leucopenia		Thrombocytopenia		Hematologic toxicity		Overall toxicity	
	G3-4/0-2	OR (95% CI)^b^	G3-4/0-2	OR (95% CI)^b^	G3-4/0-2	OR (95% CI)^b^	G3-4/0-2	OR (95% CI)^b^
Haplotype frequency								
Hap1_AAGAA	164/880	1.00	30/983	1.00	255/780	1.00	311/702	1.00
Hap2_CAAAC	92/560	0.75 (0.57−1.00)	23/608	0.97 (0.56−1.68)	142/500	0.79 (0.62−0.99)	176/459	0.77 (0.62−0.97)
Hap3_CGGAC	23/149	0.75 (0.46−1.23)	7/159	1.37 (0.60−3.12)	37/133	0.83 (0.56−1.23)	47/118	0.88 (0.61−1.28)
Hap4_CGGGC	19/66	**1.88 (1.04−3.40)**	8/75	**2.81 (1.19−6.60)**	29/55	**1.88 (1.14−3.08)**	35/49	**1.89 (1.17−3.06)**
Hap5_others	0/7	NA	0/7	NA	1/6	0.55 (0.06−4.91)	1/6	0.39 (0.04−3.51)
Diplotype frequency								
Non-Hap4 carriers	130/767	1.00	27/842	1.00	204/683	1.00	251/619	1.00
Hap4 carriers	19/64	**2.16 (1.19−3.92)**	7/74	**3.09 (1.20−7.93)**	28/54	**1.98 (1.19−3.29)**	34/48	**1.98 (1.21−3.22)**

^*a*^Haplotypes were predicted with PHASE basing on rs4979223 (A/C), rs4978536 (A/G), rs2233914 (G/A), rs10817464 (A/G) and rs10759637(A/C) that were associated with toxicity outcomes as shown in Table 2.

^*b*^Odds ratios (OR) and their 95% confidence intervals (CIs) were calculated with unconditional logistic regression analysis, with adjustment of gender, age, smoking status, ECOG performance status, TNM status, histological types, and treatment regimen.

In the further stratification analysis by demographic and clinical characteristics (Table 4), rs10759637 was associated in under-dominant model with thrombocytopenia in males (OR 5.08; 95% CI 1.62−15.94) and smoking patients (OR 4.06; 95% CI 1.40−15.11). Very similar association with thrombocytopenia was also observed for rs4979223. In alike manner, rs4978536 was associated in dominant model with thrombocytopenia in males (OR 3.16; 95% CI 1.18−8.47) and patients with adenocarcinoma (OR 3.05; 95% CI 1.26−7.37). Interestingly, in the subgroup patients with age ≤58 year or with TNM stage IV, rs10817464 consistently manifested significant association, in dominant model, with leucopenia, hematological toxicity, and overall toxicity, with ORs (95% CI) being 2.96 (1.26−6.96) and 2.88 (1.35−6.13), 2.27 (1.10−4.66) and 2.50 (1.31−4.76), 2.50 (1.25−4.96) and 2.56 (1.36−4.83), respectively. On the other hand, rs2233914 was associated with reduced overall toxicity in males (OR 0.63; 95% CI 0.44−0.90), patients treated with platinum-navelbine (OR 0.45; 95% CI 0.27−0.75) in dominant model, and in patients with adenocarcinoma (OR 0.41; 95% CI 0.21−0.81) in additive model. These data suggest that common variants of *SLC31A1* gene are associated with hematological toxicological outcomes of platinum-based chemotherapy for NSCLC patients.

**Table 4:** Stratification analysis of association between *SLC31A1* SNPs and toxicity outcomes

SNP (Wild/Variant, W/V)	Toxicity	Stratification subgroup		Genotype (WW−WV−VV)		Genetic model^a^	OR (95% CI)^b^	*P* value^b^
				G3-4	G0-2			
rs4979223 (A/C)	Thrombocytopenia	Gender	Male	2−15−2	202−285−161	Under-DOM	4.93 (1.57−15.48)	0.006
			Female	1−9−5	80−132−53	Under-DOM	1.22 (0.37−3.96)	0.744
		Smoking status	Smoker	2−13−2	161−240−128	Under-DOM	**4.44 (1.35−14.59)**	**0.014**
			Nonsmoker	1−10−5	121−175−85	Under-DOM	1.74 (0.58−5.25)	0.327
rs4978536 (A/G)	Thrombocytopenia	Gender	Male	11−8−0	507−133−11	DOM	**3.16 (1.18−8.47)**	**0.022**
			Female	9−5−1	194−62−9	DOM	1.86 (0.57−6.13)	0.305
		Histological type	AC	14−10−1	451−115−10	DOM	**3.05 (1.26−7.37)**	**0.013**
			SCC	4−1−0	147−49−8	DOM	0.60 (0.06−6.53)	0.677
rs2233914 (G/A)	Overall toxicity	Gender	Male	90−71−21	208−207−70	DOM	**0.63 (0.44−0.90)**	**0.012**
			Female	45−53−5	91−70−21	REC	**0.28 (0.09−0.86)**	**0.026**
		Histological type	AC	85−83−14	182−181−60	ADD	**0.41 (0.21−0.81)**	**0.010**
			SCC	26−24−4	80−56−20	REC	0.45 (0.13−1.61)	0.222
		Therapy regimens	Pt-navelbine	64−41−12	64−77−29	DOM	**0.45 (0.27−0.75)**	**0.002**
			Pt-gemcitabine	18−42−6	82−65−20	DOM	**2.29 (1.19−4.42)**	**0.013**
			Pt-paclitaxe	35−21−5	119−97−24	DOM	0.69 (0.37−1.28)	0.233
			Pt-docetaxel	10−15−2	21−25−12	ADD	0.12 (0.01−1.73)	0.118
rs10817464 (A/G)	Leucopenia	Age	≤58	56−10−0	404−32−2	DOM	**2.96 (1.26−6.96)**	**0.013**
			>58	74−9−0	363−30−0		1.49 (0.63−3.52)	0.369
		Smoking status	Smoker	72−12−0	448−30−1	DOM	**2.44 (1.10−5.38)**	**0.027**
			Nonsmoker	57−7−0	316−32−1	DOM	1.74 (0.66−4.58)	0.265
		TNM stage	IIIA	7−3−0	65−5−1	DOM	24.55 (1.56−387.20)	0.023
			IIIB	36−2−0	224−24−0		0.59 (0.13−2.81)	0.509
			IV	87−14−0	474−32−1		**2.88 (1.35−6.13)**	**0.006**
	Thrombocytopenia	Age	≤58	16−1−1	432−41−1	DOM	1.13 (0.23−5.46)	0.879
			>58	11−5−0	410−32−0		**6.63 (1.81−24.26)**	**0.004**
	Hematologic toxicity	Gender	Male	142−13−0	491−35−1	DOM	1.31 (0.66−2.60)	0.447
			Female	62−14−1	192−18−0	DOM	**4.19 (1.75−10.00)**	**0.001**
		Age	≤58	100−13−1	357−28−1	DOM	**2.27 (1.10−4.66)**	**0.026**
			>58	104−14−0	326−25−0		1.78 (0.85−3.72)	0.126
		Smoking status	Smoker	111−13−0	404−29−1	DOM	1.48 (0.72−3.05)	0.289
			Nonsmoker	91−14−1	277−24−0	DOM	**2.49 (1.17−5.30)**	**0.018**
		TNM stage	IIIA	10−4−0	60−4−1	DOM	7.60 (1.01−57.31)	0.049
			IIIB	57−4−0	201−21−0		0.74 (0.24−2.34)	0.612
			IV	137−19−1	418−27−0	DOM	**2.50 (1.31−4.76)**	**0.006**
	Overall toxicity	Gender	Male	167−15−0	451−33−1	DOM	1.29 (0.66−2.49)	0.456
			Female	84−18−1	168−14−0	DOM	**4.30 (1.81−10.23)**	**0.001**
		Age	≤58	124−17−1	321−24−1	DOM	**2.50 (1.25−4.96)**	**0.009**
			>58	127−16−0	298−23−0		1.68 (0.82−3.44)	0.156
		Smoking status	Smoker	130−15−0	373−27−1	DOM	1.49 (0.74−2.99)	0.261
			Nonsmoker	117−18−1	246−20−0	DOM	2.39 (1.15−4.95)	0.020
		TNM stage	IIIA	12−6−0	57−2−1	DOM	9.22 (1.49−57.07)	0.017
			IIIB	71−4−0	181−21−0		0.55 (0.18−1.71)	0.301
			IV	168−23−1	377−23−0	DOM	**2.56 (1.36−4.83)**	**0.004**
rs10759637 (A/C)	Thrombocytopenia	Gender	Male	2−15−2	207−284−160	Under-DOM	**5.08 (1.62−15.94)**	**0.005**
			Female	1−9−5	79−130−56	Under-DOM	1.26 (0.39−4.06)	0.703
		Smoking status	Smoker	2−13−2	165−238−128	Under-DOM	**4.60 (1.40−15.11)**	**0.012**
			Nonsmoker	1−10−5	121−174−87	Under-DOM	1.76 (0.59−5.30)	0.313

^*a*^For each SNP, three different genetic models (dominant, recessive and additive) were analyzed, and the model with lowest *P* values was considered the best-fitting model. Under-dominant model was also analyzed for rs4979223 and rs10759637.

^*b*^Odds ratios (OR) and their 95% confidence intervals (CIs) and *P* values were calculated with unconditional logistic regression analysis, with adjustment of gender, age, smoking status, ECOG performance status, TNM status, histological types, and treatment regimen.

We also analyzed the association between *SLC31A1* polymorphism and chemotherapy objective response. None of the eight candidate SNPs of *SLC31A1* displayed statistically significant difference in genotypic distribution between the complete or partial response group and the stable or progressive disease group (Supplementary Table 3). These results do not support genetic correlation between *SLC31A1* and objective response of platinum-based chemotherapy in the patient cohort.

### Association of SLC31A1 gene polymorphisms with survival

We measured genetic association of *SLC31A1* polymorphism with overall survival (OS) and progression-free survival (PFS) in the patient cohort with platinum-based chemotherapy by using log-rank test and Cox proportional hazards regression model (Table 5). As to the OS dataset, the log-rank test showed that rs4979223 (*P* = 0.006), rs2233914 (*P* = 0.016) and rs10759637 (*P* = 0.010) were significantly associated with survival. The median OS time of patients with heterozygous A/C of rs10759637 was significantly shorter than those patients with homozygous A/A or C/C (17.7 *vs* 20.2, *P* = 0.004). Cox proportional hazards regression analysis further showed that rs10759637 A/C was associated with increased risk of disease progression as compared with the homozygous genotype group (HR 1.24; 95% CI 1.07−1.44). In alike manner, rs4979223 heterozygous genotype was also a risk factor for OS with HR of 1.25 (1.08−1.45) and log-rank *P* value of 0.002 when assuming under-dominant model. On the contrary, rs2233914 variant A/A homozygote, in recessive model, was a beneficial factor for OS with HR of 0.73 (0.58−0.92), the median OS time of patients A/A genotype was significantly longer than those with G/G or G/A genotypes (22.3 *vs* 18.8, log-rank *P* = 0.011). As to the PFS dataset, we did not observe any association between progression-free survival and the eight candidate SNPs of *SLC31A1*.

**Table 5:** Association between *SLC31A1* SNPs and survival

		Overall survival					Progression-free survival				
SNP	Genotype	n/N^a^	MST (m)^b^	Log-rank *P*	HR (95% CI)^c^	*P*^c^	n/N d	MST (m) b	Log-rank *P*	HR (95% CI)^c^	P^c^
rs4979223	A/A	206/289	19.3	0.006	1.00 (ref)		174/271	7.5	0.292	1.00 (ref)	
	A/C	358/453	17.7		1.18 (0.99–1.41)	0.069	257/416	9.1		0.86 (0.71–1.05)	0.143
	C/C	162/227	21.0		0.88 (0.71–1.09)	0.236	126/207	10.3		0.83 (0.66–1.05)	0.126
	A/C *vs* A/A+C/C	368/516	20.0	0.002	**1.25 (1.08–1.45)**	**0.004**	300/478	9.1	0.549	0.94 (0.79–1.11)	0.452
rs4978536	A/A	551/733	19.4	0.115	1.00 (ref)		417/674	9.5	0.613	1.00 (ref)	
	A/G	163/218	17.6		1.14 (0.95–1.36)	0.165	128/203	8.3		1.11 (0.91–1.36)	0.317
	G/G	14/21	29.2		0.76 (0.45-1.31)	0.323	13/19	5.3		1.12 (0.64–1.97)	0.685
rs2233914	G/G	326/448	19.1	0.016	1.00 (ref)		268/416	7.5	0.087	1.00 (ref)	
	G/A	315/403	18.6		1.09 (0.92–1.27)	0.317	226/373	9.7		0.82 (0.69–0.99)	0.039
	A/A	87/121	22.3		0.76 (0.60–0.97)	0.029	64/107	11.1		0.78 (0.59–1.03)	0.084
	A/A *vs* G/G+G/A	641/851	18.8	0.011	**0.73 (0.58–0.92)**	**0.008**	494/789	8.7	0.200	0.86 (0.66–1.12)	0.269
rs10817464	A/A	664/886	19.0	0.520	1.00 (ref)		515/817	9.1	0.467	1.00 (ref)	
	A/G	62/84	23.5		0.89 (0.68–1.16)	0.391	42/77	9.7		0.83 (0.60–1.14)	0.249
	G/G	2/2	29.2		1.32 (0.32–5.40)	0.703	1/2	3.1		0.61 (0.08–4.44)	0.626
rs10981699	G/G	425/564	19.3	0.821	1.00 (ref)		317/515	9.5	0.264	1.00 (ref)	
	G/A	265/358	19.2		0.94 (0.80–1.10)	0.449	209/334	8.1		1.04 (0.87–1.24)	0.708
	A/A	38/50	19.4		0.96 (0.69–1.35)	0.832	32/47	6.3		1.29 (0.88–1.87)	0.192
rs10817465	A/A	393/516	19.3	0.482	1.00 (ref)		292/475	9.2	0.437	1.00 (ref)	
	A/G	283/386	19.8		0.98 (0.84–1.14)	0.782	222/355	9.2		1.02 (0.85–1.22)	0.830
	G/G	52/70	16.4		1.11 (0.82–1.50)	0.516	44/66	6.6		1.21 (0.87–1.68)	0.260
rs10513202	A/A	665/889	19.3	0.664	1.00 (ref)		501/817	9.3	0.085	1.00 (ref)	
	A/G	62/82	19.6		1.03 (0.79–1.35)	0.825	56/78	6.1		1.27 (0.96–1.69)	0.094
	G/G	0/0	NA		NA	NA	0/0	NA		NA	NA
rs10759637	A/A	209/293	19.3	0.010	1.00 (ref)		175/274	7.6	0.394	1.00 (ref)	
	A/C	356/450	17.7		1.17 (0.98–1.40)	0.075	254/413	9.2		0.88 (0.72–1.07)	0.204
	C/C	163/229	20.9		0.89 (0.72–1.10)	0.268	129/209	10.3		0.86 (0.68–1.09)	0.214
	A/C *vs* A/A+C/C	372/522	20.2	0.004	**1.24 (1.07–1.44)**	**0.005**	304/483	9.1	0.520	0.94 (0.79–1.12)	0.479

^*a*^ Numbers indicate the death event for NSCLC patients during the following-up time among all individuals in the same genotype group.

^*b*^ MST: median survival time.

^*c*^ Hazard ratios (HR) and their 95% confidence intervals (CIs) and *P* values were calculated with by multivariate Cox proportional hazards regression with adjustment for covariates.

^*d*^ Numbers indicate patients who suffered of disease progression (including death) during the following-up time among all individuals in the same genotype group.

**Figure 2:**
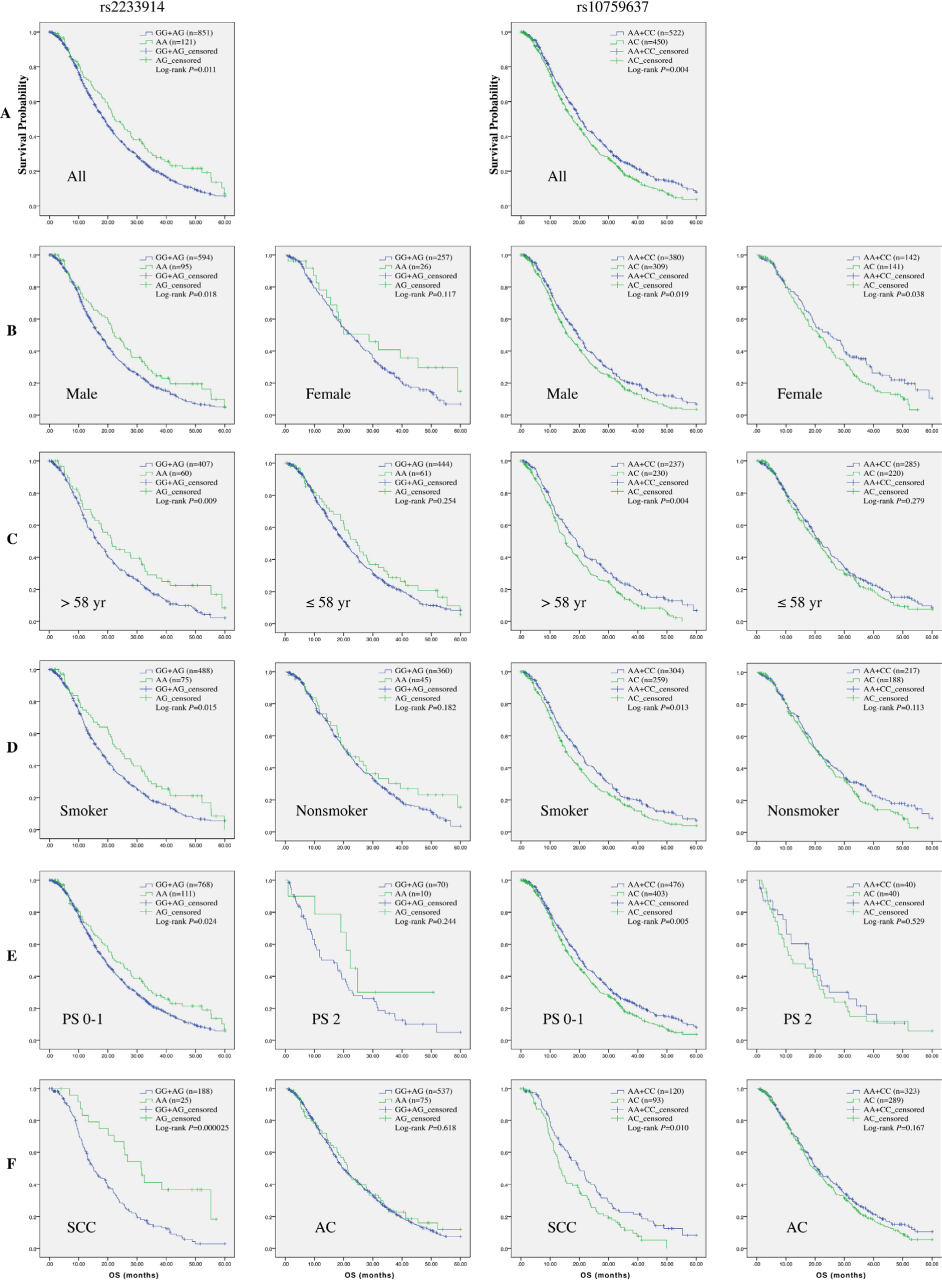
Kaplan-Meier curve of estimated overall survival for the NSCLC cohort according to *SLC31A1* polymorphisms In the entire patients (**A**) and subgroups stratified respectively with gender (**B**), age (**C**), smoking status (**D**), ECOG performance status (**E**), and histological type (**F**), the curves were plotted with SPSS software according to genotypes of two SNPs. For rs2233914, the A/A homozygote was compared to the G/G+G/A group as reference. For rs10759637, the A/C heterozygote was compared to the A/A+C/C homozygotes group as reference.

Notably, in further stratification analysis of survival data, rs4979223, rs2233914 and rs10759637 consistently manifested significant association in concordant stratification spectrum (Table 6 and Figure 2A–2F). Influence of rs10759637 heterozygous A/C genotype on OS were pronounced in male patients (log-rank *P* = 0.019; HR 1.23; 95% CI 1.04−1.47), patients older than 58 (log-rank *P* = 0.004; HR 1.38; 95% CI 1.10−1.73), ever smoker (log-rank *P* = 0.013; HR 1.28; 95% CI 1.05−1.55), patients with ECOG PS 0–1 (log-rank *P* = 0.005; HR 1.24; 95% CI 1.06−1.45), patients with squamous cell cancer (log-rank *P* = 0.010; HR 1.57; 95% CI 1.12−2.20), and patients treated with platinum-docetaxel (log-rank *P* = 0.013; HR 1.95; 95% CI 1.03−3.67), respectively. A very similar stratification spectrum with association signal was also observed for rs4979223. And also in the six subgroups of stratification, rs2233914 variant A/A homozygote, in recessive model, was beneficial modifier of OS with HRs (95% CI) being 0.75 (0.58−0.97), 0.74 (0.53−1.05), 0.73 (0.55−0.98), 0.76 (0.60−0.96), 0.38 (0.22−0.66) and 0.38 (0.17−0.87), respectively. These results clearly demonstrate that non-coding variants of *SLC31A1* are associated with overall survival of NSCLC patients with platinum-based chemotherapy.

**Table 6:** Stratification analysis of association between *SLC31A1* SNPs and overall survival

SNP (W/V, (Wild /Variant)	Stratification subgroup		Overall survival								
			n/N^a^			MST (m)^b^			Log-rank P^c^	HR (95% CI)^d^	*P*^d^
			WW	WV	VV	WW	WV	VV			
rs4979223 (A/C)	Gender	Male	150/207	248/310	128/169	18.3	15.8	21.0	0.034	**1.26 (1.06–1.50)**	**0.009**
		Female	56/82	110/143	34/58	25.1	21.4	20.3	0.049	1.19 (0.87–1.61)	0.274
	Age	≤58	112/164	164/223	83/117	21.2	20.9	22.2	0.525	1.12 (0.89–1.42)	0.346
		>58	94/125	194/230	79/110	19.1	15.2	20.0	0.003	**1.38 (1.10–1.74)**	**0.005**
	Smoking status	Smoker	121/166	211/261	99/134	18.3	15.3	21.5	0.026	**1.29 (1.07–1.57)**	**0.009**
		Nonsmoker	85/123	144/189	62/92	21.7	20.9	20.3	0.176	1.16 (0.91–1.47)	0.244
	ECOG PS	0-1	189/265	316/404	150/209	19.7	17.7	21.0	0.010	**1.26 (1.08–1.48)**	**0.003**
		2	13/19	35/41	12/18	11.8	12.4	21.8	0.372	1.15 (0.62–2.14)	0.665
	Histologic type	AC	128/181	227/292	98/138	21.3	19.1	20.9	0.236	1.13 (0.94–1.37)	0.204
		SCC	48/66	75/92	37/53	16.6	13.1	22.2	0.022	**1.51 (1.08–2.12)**	**0.017**
	Therapy regimens	Pt-navelbine	67/95	106/134	56/77	22.6	17.1	21.6	0.217	1.31 (1.00–1.71)	0.047
		Pt-gemcitabine	40/62	96/119	37/54	17.9	18.1	24.7	0.208	1.25 (0.92–1.72)	0.158
		Pt-paclitaxe	78/102	106/139	43/57	19.0	17.7	18.7	0.842	1.12 (0.85–1.48)	0.408
		Pt-docetaxel	12/16	34/40	18/27	15.9	17.4	24.3	0.013	**1.95 (1.03–3.67)**	**0.040**
rs2233914 (G/A)	Gender	Male	231/308	226/286	71/95	17.9	17.2	22.3	0.024	**0.75 (0.58–0.97)**	**0.029**
		Female	95/140	89/117	16/26	23.5	21.9	28.7	0.280	0.60 (0.34–1.08)	0.087
	Age	≤58	170/243	146/201	43/61	21.2	20.4	24.8	0.265	0.76 (0.53–1.09)	0.137
		>58	156/205	169/202	44/60	16.3	17.0	21.3	0.031	0.74 (0.53–1.05)	0.088
	Smoking status	Smoker	187/247	191/241	55/75	17.9	16.4	24.3	0.027	**0.73 (0.55–0.98)**	**0.033**
		Nonsmoker	139/201	121/159	31/45	21.2	21.0	21.3	0.380	0.74 (0.50–1.09)	0.124
	ECOG PS	0-1	296/408	279/360	81/111	19.5	18.2	22.2	0.023	**0.76 (0.60–0.96)**	**0.022**
		2	23/32	32/38	6/10	12.4	19.1	22.3	0.431	0.67 (0.26–1.72)	0.401
	Histologic type	AC	196/275	204/262	54/75	20.2	19.6	21.3	0.484	0.90 (0.67–1.21)	0.483
		SCC	81/107	64/81	16/25	15.2	14.9	31.3	0.001	**0.38 (0.22–0.66)**	**0.001**
	Therapy regimens	Pt-navelbine	100/142	96/119	33/45	19.5	17.2	22.2	0.263	0.75 (0.51–1.11)	0.148
		Pt-gemcitabine	72/103	83/106	19/27	17.9	19.8	28.7	0.231	0.67 (0.40–1.09)	0.108
		Pt-paclitaxe	116/154	89/119	23/27	19.4	18.0	16.5	0.352	1.37 (0.88–2.15)	0.164
		Pt-docetaxel	23/29	33/39	8/15	15.9	16.4	36.7	0.058	**0.38 (0.17–0.87)**	**0.021**
rs10759637 (A/C)	Gender	Male	154/212	247/309	127/168	18.3	16.0	20.9	0.059	**1.23 (1.04–1.47)**	**0.019**
		Female	55/81	109/141	36/61	25.8	21.4	20.3	0.057	1.22 (0.90–1.65)	0.193
	Age	≤58	112/165	162/220	85/120	21.2	20.9	22.2	0.557	1.13 (0.89–1.43)	0.321
		>58	97/128	194/230	78/109	19.1	15.3	19.3	0.006	**1.38 (1.10–1.73)**	**0.005**
	Smoking status	Smoker	125/170	209/259	99/134	18.3	15.7	21.5	0.035	**1.28 (1.05–1.55)**	**0.013**
		Nonsmoker	84/123	144/188	63/94	22.4	20.9	20.2	0.223	1.17 (0.92–1.48)	0.210
	ECOG PS	0-1	191/267	316/403	149/209	19.7	17.7	20.4	0.016	**1.24 (1.06–1.45)**	**0.007**
		2	14/21	34/40	13/19	11.8	12.4	21.8	0.492	1.33 (0.75–2.38)	0.335
	Histologic type	AC	131/184	224/289	99/139	19.7	19.4	20.9	0.385	1.10 (0.91–1.33)	0.307
		SCC	48/67	76/93	37/53	19.0	13.6	22.2	0.017	**1.57 (1.12–2.20)**	**0.009**
	Therapy regimens	Pt-navelbine	68/96	105/132	56/78	21.3	17.2	21.6	0.271	1.29 (0.99–1.69)	0.063
		Pt-gemcitabine	42/64	94/117	38/55	17.9	18.1	24.7	0.211	1.24 (0.91–1.70)	0.168
		Pt-paclitaxe	78/103	108/141	42/56	19.4	17.7	18.7	0.745	1.12 (0.85–1.47)	0.424
		Pt-docetaxel	12/16	34/40	18/27	15.9	17.4	24.3	0.013	**1.95 (1.03–3.67)**	**0.040**

^*a*^Numbers indicate the death event for NSCLC patients during the following-up time among all individuals in the same genotype group.

^*b*^MST: median survival time.

^*c*^Log-rank tests for association between survival and overall genotypes.

^*d*^Hazard ratios (HR) and their 95% confidence intervals (CIs) and *P* values were calculated with by multivariate Cox proportional hazards regression with adjustment for covariates. Under-dominant, recessive and under-dominant models were used to estimate the HRs for the three SNPs, respectively. For rs4979223, A/C heterozygote was compared to A/A+C/C homozygotes as reference; For rs2233914, A/A homozygote was compared to G/G+G/A group as reference. For rs10759637, A/C heterozygote was compared to A/A+C/C homozygotes as reference.

### rs10759637 at SLC31A1 3′UTR affects hsa-miR-29 mediated expression regulation

In this pharmacogenetic study, clinical outcomes such as severe thrombocytopenia hematological toxicity and overall survival of NSCLC patients with platinum-based chemotherapy were associated with linked non-coding variants of SLC31A1 gene that codes the transporter for platinum agent intake. We hypothesized that the underlying mechanism for the genetic association might be linked to functional interplay between polymorphic *cis*-elements and putative *trans*-acting factors such as transcription factor and microRNA. In order to identify putative microRNA targeting sequences in *SLC31A1* 3′UTR and assess potential effect of rs10759637 on microRNA-mediated regulation, we used the miRdSNP database and TargetScanHuman 6.2 program to screen potential microRNA binding sites at *SLC31A1* 3′UTR. Interestingly, as shown in Figure 1B, we predicted two putative binding sites, 314 bp upstream and 60 bp downstream to rs10759637, respectively, for the hsa-miR-29 family that was reported to be expressed in lung tissue and implicated in lung development and carcinogenesis [14, 15]. Further prediction of local secondary structure of *SLC31A1* mRNA 3′UTR with RNAfold program revealed that rs10759637 could alter the local mRNA secondary structure in the binding sites of hsa-miR-29abc by changing the minimum free energy from -133.40 kcal/mmol at the wild A allele state to -131.00 kcal/mmol at the variant C allele state (Figure 1C), suggesting that rs10759637 could modulate the regulatory function of hsa-miR-29 on *SLC31A1* 3′UTR. As to rs4979223 at 5′flanking region that was strictly associated with rs10759637, we did not observe putative binding sites containing it or other nearby linked SNPs in the 1000genome dataset for any transcription factor (data not shown). These results suggest that rs10759637 at *SLC31A1* 3′UTR might modulate the miRNA-mRNA interaction and affect miRNA-mediated expression regulation.

Using luciferase reporter gene assays in human bronchial epithelial cell line 16HBE, which, like lung epithelial cell, originates from primitive pluripotent pulmonary epithelial cells, we further investigated the functional modulation of rs10759637 on hsa-miR-29−*SLC31A1* 3′UTR interaction. In accordance with results of structural prediction as above, co-transfection with hsa-miR-29 member (a, b, or c) and *SLC31A1* 3′UTR construct (of rs10759637 A or C allele), as compared to the negative control microRNA mimic, consistently resulted in remarkably decreased luciferase activity, demonstrating that the hsa-miR-29 family is indeed a negative regulator for *SLC31A1* expression by targeting its 3′UTR. Interestingly, in the setting of cotransfection with hsa-miR-29 b or c, rs10759637 A/C at 3′UTR significantly affected the *cis*-regulation toward reporter expression, the variant C allele correlated with much lowered luciferase activity as compared to the wild A allele (Figure 1D, *P* < 0.05). Finally, in lung tumorous tissues from an independent cohort of patients, we analyzed the correlation between rs10759637 genotypes and transcriptional expression level for *SLC31A1* using real-time quantitative RT-PCR. Consistent with the results of structure prediction and reporter assays, we found that, as compared with the wild A/A genotype, the heterozygous A/C (*P* = 0.018) and the A/C+C/C group (*P* = 0.014) were significantly correlated with lowered expression of *SLC31A1* transcript in lung cancer tissues, suggesting that the modulation of rs10759637 on hsa-miR-29−*SLC31A1* 3′UTR interaction observed in cell line has relevance in clinical samples.

Taken together, these functional studies revealed that rs10759637 in *SLC31A1* 3′UTR could modulate the microRNA-3′UTR interaction and thereby decrease gene expression, thus proposing a possible underlying mechanism for the genetic association between *SLC31A1* polymorphism and clinical outcomes of platinum-based chemotherapy in NSCLC patients.

## DISCUSSION

In this multi-institutions based pharmacogenetic study of NSCLC patients with platinum-based chemotherapy, we found linked non-coding variants of *SLC31A1* gene, which encodes the major platinum uptake transporter, are associated with severe hematological toxicities such as thrombocytopenia and shorter overall survival. Further mechanistic analysis revealed that one SNP in 3′UTR correlated with downregulated gene expression through modulating the microRNA−3′UTR interaction. The clinically relevant and biologically functional *SLC31A1* polymorphism provides potential biomarker for outcome prediction of platinum-based chemotherapy and lung cancer management.

As a well-defined transporter for platinum import, the expression and function of SLC31A1 are involved in platinum disposition and clinical response to platinum-based chemotherapy. Chen *et al* reported that in a cohort of stage Ⅲ NSCLC patients in Chinese population receiving first-line platinum-based doublet chemotherapy, SLC31A1 protein overexpression is associated with better therapeutic response and better survival [16]. In a cohort of American patients who underwent neoadjuvant platinum-based chemotherapy, Kim *et al* also observed correlation between SLC31A1 expression in tumor size and tissue platinum concentration, undetectable protein expression and reduced drug load are associated with reduced tumor response and shorter survival time [9, 10]. Consistently, low levels of SLC31A1 mRNA are also associated with poor clinical response to platinum-based therapy in ovarian cancer patients [17]. These clinical studies demonstrate that the expression status *in situ* of SLC31A1 is a predictor for platinum-based therapy response and a significant prognostic factor for advanced NSCLC patients. However, the resectable cases only account for 10-15% of diagnosed NSCLC patients, and routine measure of SLC31A1 mRNA or protein expression in lung cancer tissues is not convenient. Thus, it is of very limited clinical utility for SLC31A1 expression as a putative biomarker of response and outcome for platinum-based chemotherapy in NSCLC patients. We here present evidences that rs10759637 in *SLC31A1* 3′UTR functionally decreased microRNA-mediated gene expression, and this common non-coding variants as well as linked SNPs were associated with severe hematological toxicities such as thrombocytopenia and shorter overall survival in the Chinese cohort of NSCLC patients with platinum-based chemotherapy. Notably, the genetic associations with survival were consistently pronounced in male patients, patients ever-smoking, older than 58, or with squamous cell carcinoma. Therefore, relevant variants of *SLC31A1* will be pertinent pharmacogenetic biomarkers, which is much more actionable than gene expression, for platinum-based therapy in NSCLC patients especially in these subgroups.

The association of rs10759637 in *SLC31A1* 3′UTR with clinical outcomes in NSCLC patients with platinum-based therapy is biologically plausible. In a screening *in silico* of potential interaction between microRNA and SNPs in 3′UTR of xenobiotic metabolism enzyme and transporter genes, Wei *et al* reported five putative miRNAs associated with rs10759637 of *SLC31A1* in liver [18]. However, we could not experimentally validate these results in reporter gene assays in 16HBE cells (data not shown). Interestingly, we predicted two putative binding sites near rs10759637 for hsa-miR-29 members, and the structure of local *SLC31A1* 3′UTR harboring these sites could be affected by rs10759637. Furthermore, the C allele, which was associated with poor outcomes of platinum-based therapy in NSCLC patients, correlated with reductions in luciferase activity in reporter assay and in *SLC31A1* transcript expression in clinical lung cancer tissues as well. Similarly, it was reported that one functional miRSNP, SNPs located at or near a microRNA binding site in 3′UTR of the target gene or in a microRNA, near the miR-24 binding site in the 3’UTR of dihydrofolate reductase gene (DHFR) interferes with miR-24 function, resulting in DHFR overexpression and methotrexate resistance [19]. A recent study also reported that the miRSNP rs1062980 may alter the expression of iron-responsive element binding protein 2 gene (IREB2) potentially through modulating the binding of miR-29a, and thereby modify risk of lung cancer [20]. The miR-29 family is expressed in lung tissues, and plays important roles in the pathogenesis of lung diseases by directly targeting genes such as those encoding extracellular matrix proteins and those associated with tissue invasion and metastasis in the contexts of fibrosis and adenocarcinoma, respectively [21, 22]. The regulation mechanism for *SLC31A1* expression is largely unknown, two transcription factors, Sp1 and hypoxia-induced factor 2α (HIF2α), have been shown to mediate its basal and inducible expression [23, 24]. We noticed that rs4979223 and rs2233914 at the at 5′flanking region of *SLC31A1* are not located at or near the *cis*-elements for Sp1 or HIF2α, their associations with toxicological phenotypes and survival could be largely due to their strong linkage disequilibrium with the functional rs10759637 at 3′UTR and genetic hitchhiking effect.

Therefore, the modulation of rs10759637 as miRSNP on miR-29−*SLC31A1* 3′UTR interaction not also proposes underlying mechanism for the association of *SLC31A1* polymorphism with clinical outcomes of platinum-based therapy, but also provides first evidence to link *SLC31A1* gene to the miR-29 regulatory network.

Another notable aspect of this study is that genotypes of rs10759637 and linked variants, as well as their combined haplotype and diplotype, were consistently associated with severe hematological toxicities especially thrombocytopenia. Thrombocytopenia is one of major common hematologic complications of cancer chemotherapy and is associated with increased morbidity, mortality and health care costs [25]. In a large cohort of solid patients (total *n* = 47159; NSCLC *n* = 7001) undergoing chemotherapy including platinum-based regimens (accounted for about 1/3 and were most common for NSCLC), NSCLC patients had the highest prevalence of severe thrombocytopenia (10.7%) cross the four major chemotherapy regimens, suggesting that the burden of thrombocytopenia remains high [26]. Although the precise mechanism for chemotherapy-induced thrombocytopenia is not well established, many cytotoxic agents including platinum are now known to cause thrombocytopenia by predominantly inducing apoptosis of the progenitors of megakaryocytes, the platelet precursor, at early stages of differentiation [27, 28]. As genetic evidence supporting this toxicological mechanism, a recent whole-exome sequencing study reported that severe thrombocytopenia in NSCLC patients treated with gemcitabine/carboplatin are associated with variant of DDX53 gene, a modulator of p53 mediated apoptosis [29]. Considering that platinum agents are intravenously administered, their biotransformation and dynamic distribution in the bloodstream are likely to be directly involved inducing hematologic toxicity. Because SLC31A1 is ubiquitously expressed in all tissues of vertebrates [30], part of administered platinum agents could be imported into all tissue cells including lung cancer cells as target, the other fraction remains bound with plasma protein in circulation [31]. In this scenario, we could cautiously postulate that, through interfering miRNA mediated expression, the naturally occurring germline SNP rs10759637 in *SLC31A1* 3′UTR might render reduced expression of transporter to all tissue cells, thus result in lowered platinum uptake in overall mass tissues, and accumulated platinum agents in bone marrow and peripheral blood plasma, and consequently be associated with platinum resistance of target tissue cells and hematologic toxicity such as thrombocytopenia.

We should cautiously point out that, in the study population of Chinese NSCLC patient cohort, we observed slight deviation from HWE for the genotypic distribution of two clinically relevant SNPs of *SLC31A1*, rs4979223 (*P* = 0.026) in 5′flanking region and rs10759637 (*P* = 0.011) in 3′UTR, with about 43 kb distance but nearly complete linkage disequilibrium, which was due to significant underrepresentation of their putatively disadvantageous heterozygotes in patients that were pharmacogenetically associated with poor outcomes such as severe thrombocytopenia and shorter survival. These HWE tests could be statistically well powered by the large sample of the patient cohort and the common allelic frequencies of the two SNPs [32]. However, in the SNP datasets for natural populations, we found that genotypic distribution of rs10759637 complies with HWE both in the overall global population (the 1000 genome dataset) and in Chinese Han population (the HapMap CHB dataset) (data not shown). The patient ascertainment was well defined, and the two common SNPs were of independent genotyping calling but are in nearly complete linkage disequilibrium. It is therefore at least possible that the HWE departure was due to ascertainment bias or genotyping errors, but instead, possible explanations could be sampling bias, underlying genetic model at the susceptibility locus, and other unknown factors in view of population genetics and demography [33]. Consistent with this scenario, rs10759637 manifested unique association with clinical outcomes in the NSCLC cohort with heterozygote disadvantage, which is also referred to as under-dominance, a genetic model where heterozygote has a lower overall fitness than either homozygote [34]. Although being rare cases, heterozygote disadvantage was also reported to account for the deviation from HWE of a common variant (c.677C>T) of the 5,10-methylenetetrahydrofolate reductase gene (*MTHFR*) in subfertile patients and its association with embryo aneuploidy [35]. However, the biological mechanism by which the *SLC31A1* rs10759637 A/C heterozygote could be disadvantage is unclear, especially considering that the variant C allele correlated with diminished gene expression. Furthermore, it has been reported of remarkable divergence in *SLC31A1* expression *in situ* as well as related drug disposition and response among populations with different ethnic ancestry. In the cohort of NSCLC patients in American population who underwent neoadjuvant platinum-based chemotherapy, Kim *et al.* reported that the African American had significantly reduced SLC31A1 expression in tumor (*P* = 0.001), lowered tissue platinum concentration (*P* = 0.009) and decreased tumor shrinkage (*P* = 0.016) as compared to Caucasians [9]. We also noticed that, in public SNP database, the low-expression-related C allele of rs10759637 is the ancestral allele that is common in African population, the high-expression-related A allele is the derived allele that is dominant in Caucasian and Chinese Han population, suggesting of significant population differentiation at this locus. These observations not only support the function mode of rs10759637 on *SLC31A1* expression, but also provide comparative and evolutionary pharmacogenetics implications for the association of *SLC31A1* polymorphism with clinical outcomes of platinum-based chemotherapy in lung cancer.

In summary, this pharmacogenetic study on advanced NSCLC patients with platinum-based chemotherapy has identified linked variants of platinum import transporter *SLC31A1* gene that were associated with severe hematological toxicities such as thrombocytopenia and shorter survival, one of which decreased gene expression through modulating microRNA-3′UTR interaction. Given that platinum-based chemotherapy is routinely used in clinical management of many types of cancers, and SLC31A1 is ubiquitously expressed in human tissues, validation of these findings in cohorts of lung cancer and other relevant types of cancer with larger sample size would be warranted in different ethnic populations.

## MATERIALS AND METHODS

### Patient recruitment and follow-up

This study encompassed in total 1004 eligible patients (Chinese Han) histologically diagnosed with stage III-IV NSCLC between March 2005 and January 2010 from five hospitals in the East of China: Shanghai Chest Hospital, Shanghai Zhongshan Hospital, Shanghai Changhai Hospital, Shanghai Changzheng Hospital, and Cancer Hospital of Jiangsu Province. The recruitment criteria for enrolling eligible patients and their demographic characteristics such as gender, age at diagnosis, smoking status, ECOG performance status, clinical TNM stage, and tumor histological type were described in detail in our previous reports [36–39]. No statistically significant difference was observed in the distribution of demographic features among the patients from the six hospitals (*P*_gender_ = 0.698, *P*_age_ = 0.321). In addition, previously prepared lung cancer tissues from 47 surgically resectable patients recruited at Shanghai Changzheng Hospital were used for gene expression analyses [40].

The patients enrolled in the pharmacogenetic study were inoperable and received first-line, platinum-based chemotherapy (no prior surgery, radiotherapy, or concurrent chemoradiotherapy). The chemotherapeutic regimens were as follows: either cisplatin (75 mg/m^2^) or carboplatin (at an area under the curve 5), both administered on day 1 every 3 weeks, in combination with navelbine (25 mg/m^2^) on days 1 and 8 every 3 weeks, or gemcitabine (1250 mg/m^2^) on days 1 and 8 every 3 weeks, or paclitaxel (175 mg/m^2^) on day 1 every 3 weeks, or docetaxel (75 mg/m^2^) on day 1 every 3 weeks. A few patients received other platinum-based treatment (*n* = 49). All chemotherapeutic drugs were administered intravenously, and all treatments lasted for 2 to 6 cycles.

Clinical outcomes including toxicities, responses and survival were assessed in this study. The incidence of grade 3 or 4 chemotherapy toxicity was assessed for the patients from the end of the first two cycles of treatment according to the Common Terminology Criteria for Adverse Events version 3.0 (CTCAE v3.0), including overall toxicity, gastrointestinal toxicity (nausea and vomiting), and hematologic toxicity (leucopenia, neutropenia, anemia, and thrombocytopenia) [41]. No grade 5 toxicity (death) was observed. Responses to platinum-based chemotherapy were evaluated after the first two cycles of the course, which are classified into four categories namely complete response (CR), partial response (PR), stable disease (SD) and progressive disease (PD) according to the Response Evaluation Criteria in Solid Tumors (RECIST) guidelines version 1.0 [42]. Object response rate (ORR) is defined as the percentage of patients with CR or PR. Survival data were collected from several sources including follow-up calls, the Social Security Death Index, and clinical medical records of inpatient and outpatient. Progression-free survival (PFS) was calculated from the date of chemotherapy beginning to the date of disease progression or death (whichever occurred first) or the last progression-free follow-up. Overall survival (OS) was calculated from the date of chemotherapy beginning to the date of death. The research assistants who performed the genotyping assays were blinded to the clinical state of patients and the clinical investigators were blinded to the genotypic state of patients. The study protocol was approved by the Ethical Review Committees of Fudan University School of Life Sciences and the participating hospitals, and written informed consent was obtained from each subjects.

### SNPs selection and genotyping

Eight polymorphisms were selected by an approach combining both tagging and potentially functional SNPs of *SLC31A1* gene. The tagging SNPs were screened from the Han Chinese in Beijing (CHB) population dataset of the HapMap database (http://www.hapmap.org) using a minor allele frequency (MAF) cutoff of 0.05 and a correlation coefficient (*r*^2^) threshold of 0.8. Genomic DNA was extracted from whole blood using the QIAamp DNA Maxi Kit (Qiagen GmbH, Hilden, Germany). Genotyping was performed using iSelect HD BeadChip (Illumina, San Diego, CA, USA) with the following quality-control criteria: genotyping call rate of SNP >0.95, GenCall score >0.2, and *P* value of Hardy-Weinberg equilibrium (HWE) >0.01. Genotyping of one SNP (rs10759637) at the 3′UTR for lung cancer tissues of the 47 surgically resectable patients was conducted using PCR-direct sequencing with forward primer 5′-GGAGAGCAAGGAATGTGGACT-3′ and the reverse primer 5′-GCAGCTCATGCTGAGATTTCTA-3′.

### *In silico* prediction of microRNA binding sites at the 3′UTR of *SLC31A1*

In order to identify putative microRNA targeting sequences in *SLC31A1* 3′UTR, and assess potential effect of rs10759637 on microRNA-mediated regulation, we used the miRdSNP database (http://mirdsnp.ccr.buffalo.edu/index.php) and TargetScanHuman 6.2 program (http://www.targetscan.org/) to screen microRNA binding sites, and used RNAfold (http://rna.tbi.univie.ac.at/) to predict the local secondary structure of the *SLC31A1* mRNA.

### Luciferase reporter assays

We performed luciferase reporter assays to characterize the effect of rs10759637 at *SLC31A1* 3′UTR on microRNA-mediated regulation. We constructed reporter gene vectors for rs10759637 by amplifying the target sequence about 1200bp around rs10759637 at *SLC31A1* 3′UTR from human genomic DNA, with the forward primer 5′-CCGCTCGAGTCTACCAGTCATGGGCCAGAAGG-3′ and the reverse primer 5′-ATTTGCGGCCGCGCTTCATCCTTTGGCAGCCAGTC-3′, and after digestion with *Xho* I and *Not* I, cloning into the psiCHECK2 vector (Promega, Madison, WI, USA) near 3′UTR of *Renilla* luciferase. Reporter construct for rs10759637 variant was made through point mutation (C/A) with primeSTAR using primers 5′-CAGCCTGTTGTCTCAAGCCAAGGAGTC-3′ (forward) and 5′-GACTCCTTGGCTTGAGACAACAGGCTG-3′ (reverse). Empty vector psiCHECK2 was used as negative control. Three microRNA mimics (hsa-miR-29a, 5′- UAGCACCAUCUGAAAUCGGUUA-3′; hsa-miR-29b, 5′-UAGCACCAUUUGAAAUCAGUGUU-3′ and hsa-miR-29c, 5′-UAGCACCAUUUGAAAUCGGUUA-3′) and one non-specific microRNA (miR-NC, 5′-UUG UACUACACAAAAGUACUG-3′) as negative control were synthetized from GenePharma, Shanghai China. Human bronchial epithelial cell line 16HBE (1.0 × 10^5^ cells) was cotransfected with wild or variant rs10759637 report vector (400 ng) and microRNA (40pmol miRNA) using Lipofectamine^™^ 2000 (Invitrogen). Luciferase activity was measured with Dual-Luciferase Reporter Assay System (Promega) on Envision 2104 Multilabel Reader (PerkinElmer). Every treatment was set three replications and three independent experiments were performed.

### Real-time quantitative RT-PCR

We quantified transcript level of *SLC31A1* in lung cancer tissues from 47 patients using real-time quantitative RT-PCR assay. Total RNA was extracted from tumorous lung tissues with RNeasy Plus Mini Kit (QIAGEN, Hilden, Germany) and was then reverse transcribed into cDNA with PrimeScript RT Master Mix (TaKaRa, Dalian, China) using primers 5′-GAGAGAGCCTGCTGCGTAAG-3′ (forward) and 5′-AATGCAGAGGTACCCGTTGT-3′ (reverse). qRT-PCR was performed with SYBR^®^ Premix Ex Taq Kit (Takara) on an Applied Biosystems 7900HT. We measured the expression of β-actin as internal control using primers 5′-CAGAGCCTCGCCTTTGCC-3′ (forward) and 5′-ATGCCGGAGCCGTTGTCG-3′ (reserve). Every sample was set in triplicate. Expression of *SLC31A1* was normalized to β-actin with **∆∆**C_T_ method.

### Statistical analysis

We used Pearson χ^2^ tests to examine deviations of genotype frequencies from those expected under HWE, and to test differences in the distributions of genotypes between patient groups with divergent clinical phenotypes or outcomes. We used the PHASE version 2.1 program to estimate haplotypes from the genotype data [43], and constructed the linkage disequilibrium (LD) plots using Haploview (http://www.broadinstitute.org/haploview), in which pairwise linkage disequilibrium relations among SNPs were examined using *D*′ and *r*^2^. We measured the association between genetic variants and dichotomous clinical phenotypes or outcomes by calculating odds ratios (OR) and their 95% confidence intervals (CIs) in unconditional logistic regression analysis, with adjustment of gender, age, smoking status, ECOG performance status, TNM status, histological types, and treatment regimen. Haplotype-based genetic association analysis was performed using Haplo.stats package in R-plus (available at http://cran.r-project.org/web/packages/haplo.stats.index.html; Version: 1.6.8). For each SNP, three different genetic models (dominant, recessive and additive) were analyzed, and the model with lowest *P* values was considered the best-fitting model. Under-dominant model was also analyzed for rs4979223 and rs10759637. The association between genetic variant and survival data (overall survival and progression-free-survival) was calculated by log-rank test with adjustment for covariates. The multivariate Cox proportional hazards regression was used for calculating the hazard ratios (HR) and 95% CI, Kaplan-Meier method was used to plot survival curve. All the statistical analysis was performed by SPSS (version 20). We use the two-side test for all *P* values. A *P* values < 0.05 was considered statistically significant. To account for the issue of multiple testing of SNPs, we used SNPSpD to correct the significance threshold taking into account LD between polymorphisms [44].

## SUPPLEMENTARY MATERIALS TABLES





## Abbreviations

3′UTR: 3′untranslated region
AC: adenocarcinoma
CI: confidence interval
CR: complete response
ECOG PS: Eastern Cooperative Oncology Group performance status
HWE: Hardy-Weinberg equilibrium
HR: hazard ratio
LD: linkage disequilibrium
NSCLC: non-small-cell lung cancer
OR: odds ratio
OS: overall survival
PD: progressive disease
PFS: progression-free survival
PR: partial response
SCC: squamous cell carcinoma
SD: stable disease
SLC31A1: solute carrier family 31 member 1
