# Supplementary material for: Genetic polymorphism of SLC31A1 is associated with clinical outcomes of platinum-based chemotherapy in non-small-cell lung cancer patients through modulating microRNA-mediated regulation
Source: Oncotarget. 2018 May 8;9(35):23860–77. doi: 10.18632/oncotarget.24794 (PMC5963629; doi:10.18632/oncotarget.24794)
Supplement: Supplementary file 1 [file oncotarget-09-23860-s001.pdf]

Genetic polymorphism of *SLC31A1* is associated with clinical outcomes of platinum-based chemotherapy in non-small-cell lung cancer patients through modulating microRNA-mediated regulation

SUPPLEMENTARY MATERIALS

Supplementary Table 1: *SLC31A1* tag and potentially functional SNPs genotyped in the cohort of NSCLC patients

Reference SNP	Ch9 position	Genic location	Allele (Wild/ Variant)	Genotyping rate (%)	Minor allele frequency	*P* value for HWE[a]
rs4979223	113219836	5′flanking region	A/C	99.7	0.468	0.026
rs4978536	113220123	5′flanking region	A/G	100.0	0.133	0.272
rs2233914	113221260	5′flanking region	G/A	100.0	0.333	0.039
rs10817464	113230217	intron	A/G	100.0	0.045	1.000
rs10981699	113233621	intron	G/A	100.0	0.236	0.431
rs10817465	113243503	intron	A/G	100.0	0.270	0.873
rs10513202	113262679	3′UTR	A/G	99.9	0.042	0.253
rs10759637	113262744	3′UTR	A/C	100.0	0.467	0.011

[a]Pearson $\chi 2$ tests for deviation from Hardy-Weinberg equilibrium (HWE).

Supplementary Table 2: Genotypic distribution of SLC31A1 SNPs between NSCLC patients with mild or severe toxicological outcomes. See Supplementary_Table_2

Supplementary Table 3: Association between SLC31A1 SNPs and objective response rate

Ref SNP	Genotype	Response (CR+PR/SD+PD)	*P* value[a]
rs4979223	A/A	58/230	0.531
	A/C	78/377	
	C/C	39/190	
rs4978536	A/A	127/608	0.065
	A/G	42/176	
	G/G	8/14	
rs2233914	G/G	95/352	0.052
	G/A	66/341	
	A/A	16/105	
rs10817464	A/A	160/730	0.502
	G/A	16/67	
	G/G	1/1	
rs10981699	G/G	97/466	0.519
	G/A	72/289	
	A/A	8/43	
rs10817465	A/A	84/434	0.216
	A/G	77/309	
	G/G	16/55	
rs10513202	A/A	155/735	0.046
	A/G	22/62	
	G/G	0/0	
rs10759637	A/A	60/232	0.424
	A/C	79/373	
	C/C	38/193	

[a]*P* values of Pearson χ^2 tests.
