# Supplementary material for: Genetic polymorphism of SLC31A1 is associated with clinical outcomes of platinum-based chemotherapy in non-small-cell lung cancer patients through modulating microRNA-mediated regulation
Source: Oncotarget. 2018 May 8;9(35):23860–77. doi: 10.18632/oncotarget.24794 (PMC5963629; doi:10.18632/oncotarget.24794)
Supplement: Supplementary file 2 [file oncotarget-09-23860-s002.docx]

| Toxicological phenotype  and grade (G) | | SNP genotype (Wild/Variant, WW–WV–VV) | | | | | | | |
| --- | --- | --- | --- | --- | --- | --- | --- | --- | --- |
|  |  | rs4979223 (A/C) | rs4978536 (A/G) | rs2233914 (G/A) | rs10817464 (A/G) | rs10981699 (G/A) | rs10817465 (A/G) | rs10513202 (A/G) | rs10759637 (A/C) |
| Gastrointestinal toxicity | G0-2 | 260–413–208 | 670–194–20 | 402–371–111 | 809–73–2 | 513–326–45 | 469–348–67 | 807–76–0 | 264–410–210 |
|  | G3-4 | 26–35–19 | 59–20–1 | 37–35–8 | 70–10–0 | 43–32–5 | 44–34–2 | 74–6–0 | 26–35–19 |
|  | *P* value *^a^* | 0.829 | 0.707 | 0.797 | 0.339 | 0.735 | 0.237 | 0.734 | 0.870 |
| Hematologic toxicity | G0-2 | 224–331–180 | 556–153–18 | 334–306–97 | 683–53–1 | 433–265–39 | 400–280–57 | 670–66–0 | 227–329–181 |
|  | G3-4 | 68–118–45 | 170–58–4 | 111–100–21 | 204–27–1 | 125–97–10 | 115–105–12 | 217–15–0 | 69–117–46 |
|  | *P* value *^a^* | 0.188 | 0.342 | 0.247 | 0.088 | 0.262 | 0.095 | 0.230 | 0.219 |
| Anemia | G0-2 | 278–421–213 | 695–200–20 | 421–383–111 | 840–74–1 | 525–342–48 | 486–365–64 | 835–79–0 | 282–418–215 |
|  | G3-4 | 7–16–6 | 23–5–1 | 11–15–3 | 25–3–1 | 17–12–0 | 16–10–3 | 29–0–0 | 7–16–6 |
|  | *P* value *^a^* | 0.622 | 0.873 | 0.570 | 0.046 | 0.438 | 0.714 | 0.163 | 0.590 |
| Leukopenia | G0-2 | 251–378–200 | 634–178–19 | 377–348–106 | 767–62–2 | 483–306–42 | 447–321–63 | 759–71–9 | 254–375–202 |
|  | G3-4 | 45–72–31 | 110–36–3 | 73–60–16 | 130–19–0 | 81–61–7 | 74–68–7 | 138–11–0 | 46–72–31 |
|  | *P* value *^a^* | 0.673 | 0.749 | 0.653 | 0.069 | 0.633 | 0.179 | 0.635 | 0.625 |
| Neutropenia | G0-2 | 247–374–196 | 630–172–18 | 369–346–105 | 755–64–1 | 475–304–41 | 444–316–60 | 747–72–0 | 251–372–197 |
|  | G3-4 | 37–56–22 | 83–29–3 | 59–47–9 | 102–12–1 | 64–44–7 | 55–52–8 | 107–8–0 | 37–55–23 |
|  | *P* value *^a^* | 0.515 | 0.547 | 0.227 | 0.137 | 0.835 | 0.384 | 0.510 | 0.635 |
| Thrombocytopenia | G0-2 | 282–417–214 | 701–195–20 | 422–380–114 | 842–73–1 | 523–346–47 | 489–363–64 | 839–76–0 | 286–414–216 |
|  | G3-4 | 3–24–7 | 20–13–1 | 12–21–1 | 27–6–1 | 22–11–1 | 15–16–3 | 31–3–0 | 3–24–7 |
|  | *P* value *^a^* | **0.007** | 0.063 | 0.039 | **0.006** | 0.638 | 0.565 | 0.757 | **0.006** |
| Overall toxicity | G0-2 | 199–303–163 | 515–136–16 | 299–277–91 | 619–47–1 | 394–237–36 | 366–247–54 | 606–60–0 | 202–301–164 |
|  | G3-4 | 85–140–59 | 208–72–5 | 135–124–26 | 251–33–1 | 154–118–13 | 143–128–14 | 265–20–0 | 86–139–60 |
|  | *P* value *^a^* | 0.410 | 0.221 | 0.150 | 0.050 | 0.224 | 0.033 | 0.311 | 0.441 |

*^a^ P* values of Pearson χ^2^ tests.
